# Nanopore Sequencing for Characterization of HIV-1 Recombinant Forms

**DOI:** 10.1128/spectrum.01507-22

**Published:** 2022-07-27

**Authors:** Mikiko Mori, Hirotaka Ode, Mai Kubota, Yoshihiro Nakata, Takaaki Kasahara, Urara Shigemi, Reiko Okazaki, Masakazu Matsuda, Kazuhiro Matsuoka, Atsuko Sugimoto, Atsuko Hachiya, Mayumi Imahashi, Yoshiyuki Yokomaku, Yasumasa Iwatani

**Affiliations:** a Clinical Research Center, National Hospital Organization Nagoya Medical Center, Nagoya, Japan; b Division of Basic Medicine, Nagoya University Graduate School of Medicine, Nagoya, Japan; Kumamoto University

**Keywords:** HIV-1, nanopore sequencing, recombinant form, dual infection, genetic diversity, molecular epidemiology, drug resistance

## Abstract

High genetic diversity, including the emergence of recombinant forms (RFs), is one of the most prominent features of human immunodeficiency virus type 1 (HIV-1). Conventional detection of HIV-1 RFs requires pretreatments, i.e., cloning or single-genome amplification, to distinguish them from dual- or multiple-infection variants. However, these processes are time-consuming and labor-intensive. Here, we constructed a new nanopore sequencing-based platform that enables us to obtain distinctive genetic information for intersubtype RFs and dual-infection HIV-1 variants by using amplicons of HIV-1 near-full-length genomes or two overlapping half-length genome fragments. Repeated benchmark tests of HIV-1 proviral DNA revealed consensus sequence inference with a reduced error rate, allowing us to obtain sufficiently accurate sequence data. In addition, we applied the platform for sequence analyses of 9 clinical samples with suspected HIV-1 RF infection or dual infection according to Sanger sequencing-based genotyping tests for HIV-1 drug resistance. For each RF infection case, replicated analyses involving our nanopore sequencing-based platform consistently produced long consecutive analogous consensus sequences with mosaic genomic structures consisting of two different subtypes. In contrast, we detected multiple heterologous sequences in each dual-infection case. These results demonstrate that our new nanopore sequencing platform is applicable to identify the full-length HIV-1 genome structure of intersubtype RFs as well as dual-infection heterologous HIV-1. Since the genetic diversity of HIV-1 continues to gradually increase, this system will help accelerate full-length genome analysis and molecular epidemiological surveillance for HIV-1.

**IMPORTANCE** HIV-1 is characterized by large genetic differences, including HIV-1 recombinant forms (RFs). Conventional genetic analyses require time-consuming pretreatments, i.e., cloning or single-genome amplification, to distinguish RFs from dual- or multiple-infection cases. In this study, we developed a new analytical system for HIV-1 sequence data obtained by nanopore sequencing. The error rate of this method was reduced to ~0.06%. We applied this system for sequence analyses of 9 clinical samples with suspected HIV-1 RF infection or dual infection, which were extracted from 373 cases of HIV patients based on our retrospective analysis of HIV-1 drug resistance genotyping test results. We found that our new nanopore sequencing platform is applicable to identify the full-length HIV-1 genome structure of intersubtype RFs as well as dual-infection heterologous HIV-1. Our protocol will be useful for epidemiological surveillance to examine HIV-1 transmission as well as for genotypic tests of HIV-1 drug resistance in clinical settings.

## INTRODUCTION

One of the key genetic features of HIV-1 is high genetic diversity due to its high mutation rate of reverse transcriptase and its recombination-prone nature (1–3). Hence, the HIV-1 genome shows within- and between-host genomic diversity. HIV-1 is classified into 4 groups (M, N, O, and P), with group M further divided into 10 major subtypes (A to D, F to H, and J to L). Additionally, to date, 118 circulating recombinant forms (CRFs) have also been identified according to the Los Alamos HIV sequence database (4), and unique recombinant forms (URFs) without evident transmission have also been found (5). Moreover, complex second- or third-generation CRFs resulting from further recombination of existing CRFs (6, 7) and more diversified genotypes of HIV-1 have been observed over time (7–9). A recent study reported that CRFs and URFs account for 16.7% (including CRF01_AE and CRF02_AG) and 6.1% of HIV-1 infections, respectively (10).

The emergence of intersubtype RFs results from dual or multiple infections of different HIV-1 subtypes within an individual (11). In particular, patients in populations at high risk for HIV-1 have a greater probability of generating intersubtype HIV-1 RFs since an initial HIV-1 infection does not drive an efficient immune response against a subsequent infection (12–14). Several case reports have shown that the incidence rates of reinfection with a second HIV-1 variant are approximately 0 to 7.7% per year (12, 15). The recombination of HIV-1 may also impact many aspects of the HIV-1 pandemic, including viral diversity and fitness, drug resistance, immunological escape, and disease progression, as well as the diagnostic accuracy of serological and molecular assays. Clarification of HIV-1 genetic features is important for understanding the origin, transmission, and prevalence of this virus. Therefore, we need to pay more attention to the emergence of HIV-1 RFs or multiple-infection viruses in high-risk populations. The rapid increase and large diversity of HIV-1 recombinants pose great challenges to the prevention and surveillance of HIV infection.

To date, RFs have been identified conventionally by Sanger or short-read next-generation sequencing of HIV-1 genomes, which results in patchworks of genetic information among subtypes and/or variants. Pretreatments such as cloning or single-genome amplification are required to distinguish recombinants in dual- (or multiple)-infection cases (16–18). However, these pretreatments are labor-intensive and time-consuming (19, 20). Recently, long-read sequencing technologies have been improved. One such long-read technology is Oxford Nanopore sequencing, which provides sequence information for long DNA fragments (tens to hundreds of kilobases) over short time periods. Nanopore sequencing technologies have already been applied in pathogen surveillance for Ebola, Zika, coronavirus disease 2019 (COVID-19), and other outbreaks (21–25). In fact, a recent study by Wright et al. showed a new application protocol of nanopore sequencing to analyze HIV-1 proviral DNA variants (26). However, a pretreatment process of limiting dilution is required for this method. Therefore, we sought to establish a nanopore sequencing method that does not need such limiting dilution processes by developing analytical processes in consensus sequence estimation and to inspect our platform for HIV that differentially characterizes intersubtype RFs and dual-infection HIV-1 variants by using clinical samples. The study may highlight the broad utility of nanopore sequencing for near-full-length genomes as part of HIV surveillance.

## RESULTS

### Error rates of nanopore sequencing.

We first measured the error rates of our nanopore sequencing protocol by using an HIV-1 molecular clone, pNL4-3 (27). Nanopore sequencing was employed for amplicons of the near-full-length genome (the *gag-nef* region) and two half genomes (the *gag-pol* and *env-nef* regions). Error rates (%) were calculated as the number of erroneous nucleotide substitutions, insertions, or deletions divided by the total nucleotide number of the reads. When 2,500 raw read sequences were examined, the error rates per read were relatively high: substitutions (1.9 to 2.4%), insertions (1.4 to 2.8%), and deletions (1.7 to 3.0%) (see Fig. S2 in the supplemental material). The sum of the error rates for one raw read ranged from 5.8 to 7.0%, which appears to be equivalent to that observed in the other analyses of RNA virus genomes and the human RNA transcriptome (24, 28, 29). To compensate for these error rates of raw reads, we constructed a consensus sequence(s) from multiple raw reads for further analysis. When consensus sequences were constructed from 10 different sets of 250 raw reads, the total error rate of the consensus sequence(s) was improved to 0.011 to 0.056% (Fig. S2). We identified three error-prone positions in the consensus sequences: two erroneous insertions in homopolymer regions (corresponding to nucleotide [nt] positions ~840 and ~4795 in the reference HXB2 sequence) and one short tandem repeat region (nt position ~7745). These sequence patterns are regarded as error-prone motifs in the nanopore sequencing of bacterial and human DNAs, as previously reported (30).

### Nanopore sequencing analysis of the HIV-1 RFs in clinical specimens.

**FIG 1:**
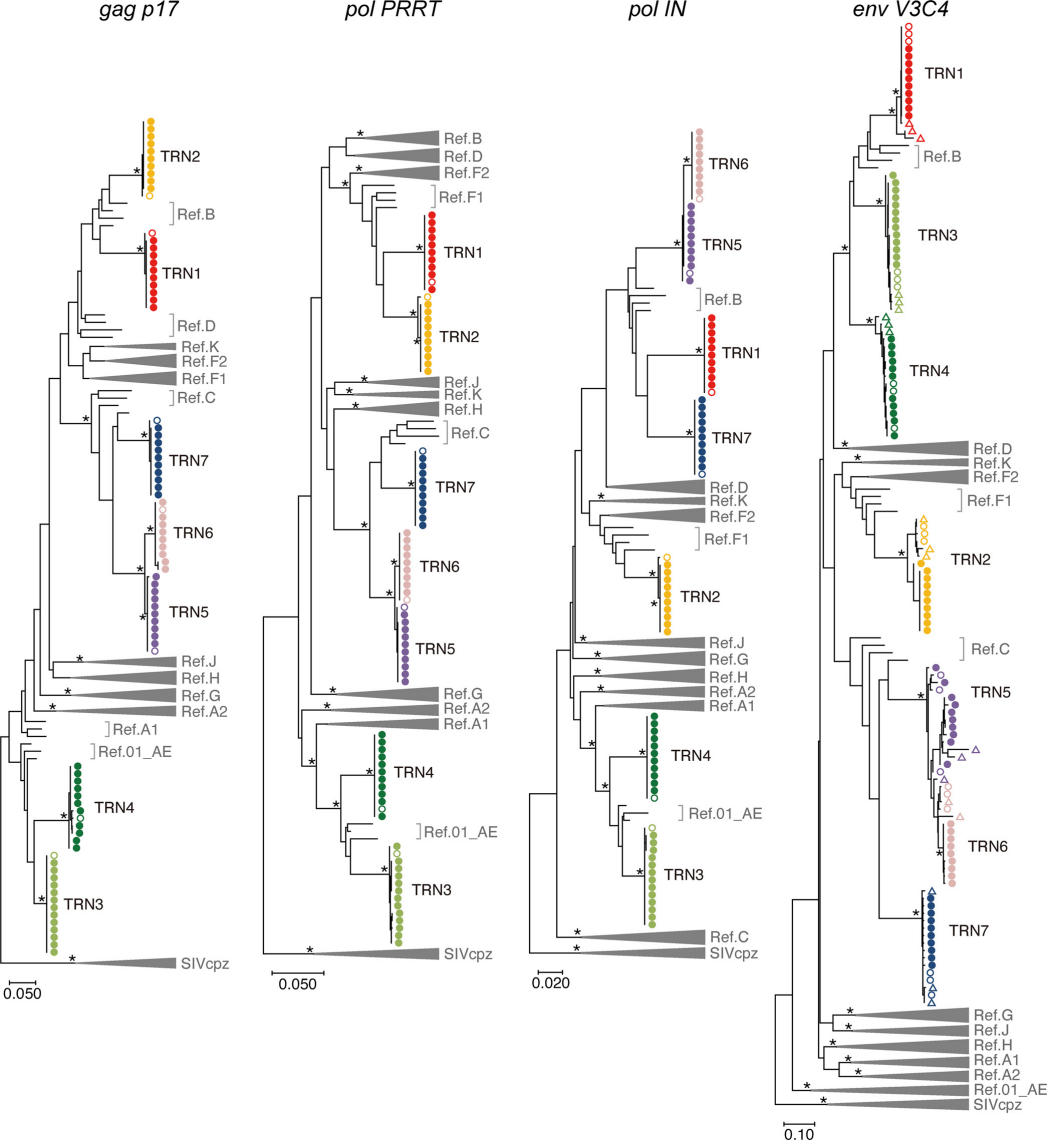
Phylogenies of the RF sequences identified by nanopore sequencing and Sanger sequencing. Maximum likelihood trees with the GTR model are shown for four regions: *gag p17* (positions 790 to 1185 of HXB2), *pol PRRT* (2253 to 3269), *pol IN* (4230 to 5093), and *env V3C4* (7114 to 7589). The reference sequences (Ref.) of HIV-1 major subtypes (A1, A2, B, C, D, F1, F2, G, H, J, K, and CRF01_AE) and three SIVcpz strains are indicated in the tree. The three SIVcpz sequences are used as outliers (GenBank no. DQ373064, DQ373063, and EF535994 for SIVcpzLB7, SIVcpzMB66, and SIVcpzMB897, respectively). Bootstrap values were calculated by 500 replicates. Branches with bootstrap values of at least 0.95 are highlighted with asterisks. The viral full-genome sequences (closed circles) identified by nanopore sequencing and the viral RNA (open circles) and proviral DNA sequences (triangles) identified by Sanger sequencing are indicated.

Based on our retrospective analysis of HIV-1 drug resistance genotyping test results (373 cases), we found 9 cases in which HIV-1 carried discordant subtype fragments in four genomic regions (*gag p17*, *pol PRRT*, *pol IN*, and *env V3C4* regions) tested for drug resistance. These 9 cases presented a high viral load in plasma. Detailed information on the samples is presented in Table 1. Of the 9 cases, 7 (sample IDs TRN1 to 7) were postulated to be HIV-1 RF infections, and the others (TRN8 and 9) were dual infections according to Sanger sequencing chromatograms with double peaks in the tested regions for drug resistance. First, nanopore sequencing was employed for HIV-1 genomes in 7 plasma samples (TRN1- to 7), which were presumably RF samples. Full-genome consensus sequences of each sample were determined by nanopore sequencing and then compared with the Sanger-based sequences in the four genomic regions. The resultant sequences obtained from these two methods displayed concordant patterns, especially in the *gag p17*, *pol PRRT*, and *pol IN* regions (Fig. S3). Similar results were also observed in phylogenetic tree analyses of these regions (Fig. 1). On the trees, the estimated consensus sequences from the nanopore data were clustered with each other as well as with the corresponding sequences generated by Sanger sequencing. In three cases (TRN2, 5, and 6), there were different sequences in the *env V3C4* region between the Sanger and nanopore sequences (Fig. S3). This is most likely due to within-host *env* sequence diversity, especially in a variable region of *env*. This explanation is corroborated by phylogenetic analysis data demonstrating that the *env V3C4* sequences for each case (TRN2, 5, and 6) formed one identical cluster with long genetic distances, regardless of the sequencing method used (Fig. 1). The samples of RF cases that we examined in this study included one heterosexual transmission pair (TRN5 and TRN6). As shown in Fig. 1, the consensus sequences of the respective cases were genetically similar to each other, although they were separable into different clusters on the genetic tree. Genetic diversity at *env V3C4* was higher for TRN5 than for TRN6, suggesting longer infection periods in the case of TRN5 and transmission from TRN5 to TRN6. The different infection periods between TRN5 and TRN6 inferred on the basis of genetic diversity are consistent with findings from clinical interviews. These results suggest that the consensus sequence from nanopore data is sufficiently accurate to determine phylogenetic relationships and perform subtype classification.

**TABLE 1:** Patient information and HIV-1 subtypes analyzed in this study

Sample ID	Age group	Gender	Nationality	Estimated location of infection	Estimated transmission route^*a*^	CD4^+^ cell counts (cells/μL)	Plasma viral load (copies/mL)	HIV-1 subtype in coding region of^*b*^			
								*gag p17*	*pol PRRT*	*pol IN*	*env V3C4*
TRN1	20s	M	Brazil	Brazil	MSM	219	11,500	B	F	B	B
TRN2	20s	F	Brazil	Unclear	Hetero	303	47,800	B	F	F	F
TRN3	30s	M	Philippines	Japan	MSM	368	57,600	01_AE	01_AE	01_AE	B
TRN4	50s	M	Japan	Japan or Thailand	MSM	28	1,230,000	01_AE	01_AE	01_AE	B
TRN5	30s	M	Brazil	Brazil	Hetero	396	54,700	C	C	B	C
TRN6	30s	F	Brazil	Brazil	Hetero	229	8,130	C	C	B	C
TRN7	30s	M	Brazil	Japan	MSM	285	32,400	C	C	B	C
TRN8	30s	M	Japan	Japan	Hetero	761	21,000	B	B	B	AE/B
TRN9	20s	M	Japan	Japan or China	MSM	229	40,400	C	01_AE^*c*^	01_AE^*c*^	C

a MSM, men who have sex with men; Hetero, heterosexual.

b The subtype information was obtained from the data of the Sanger-based drug resistance test.

c Excess numbers of mixed bases were detected in the drug resistance test.

**FIG 2:**
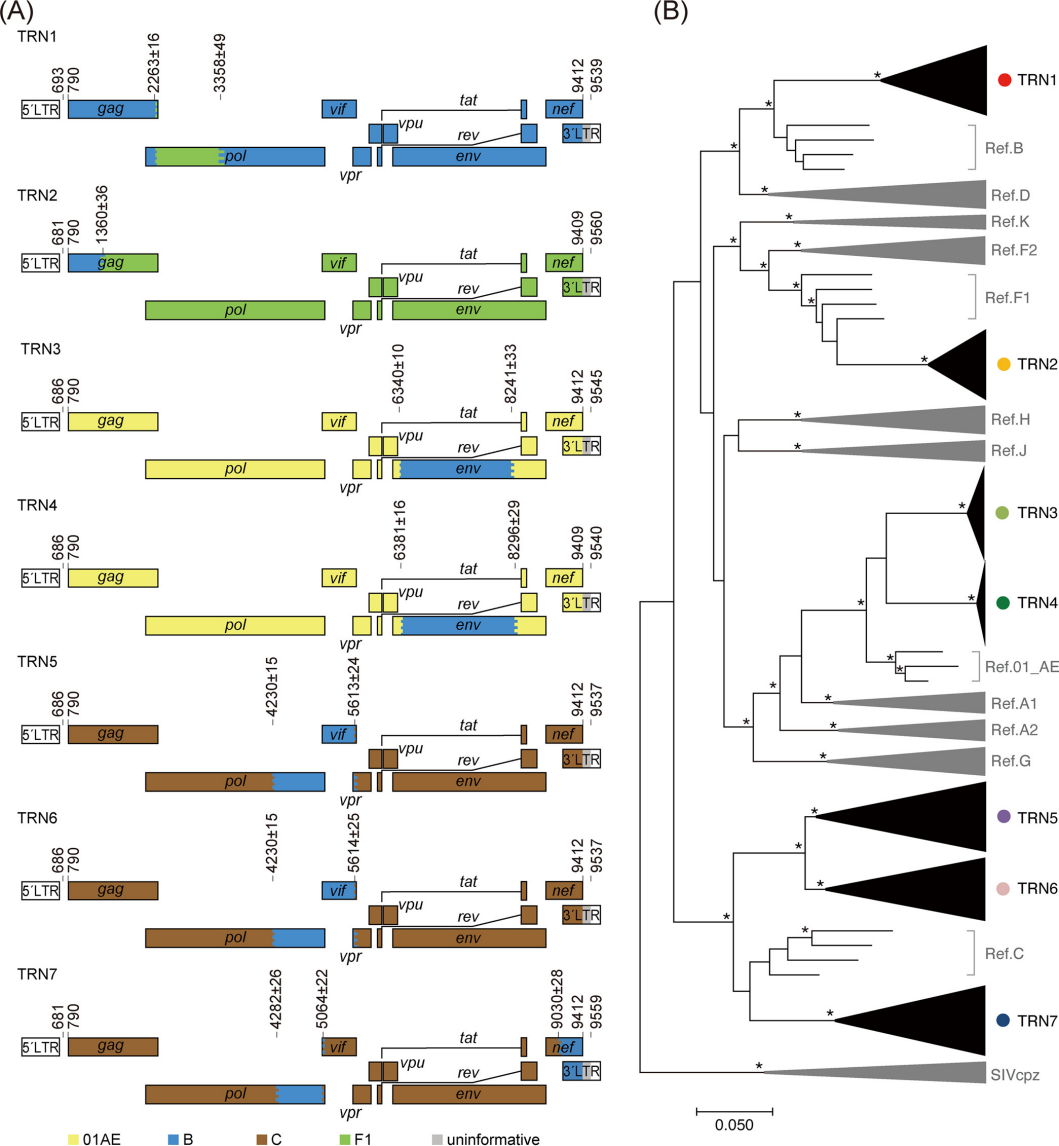
Characterization of the HIV-1 RFs. (A) Genome maps of the RFs. The numbers on each map denote the nucleotide positions of the breakpoints according to the HXB2 reference numbering system. The colored stripes in the maps indicate the intervals where the recombination breakpoints are estimated to be located. (B) Maximum likelihood tree for pangenomic consensus sequences obtained by nanopore sequencing and complete genomes of the major subtype references. The three sequences of SIVcpz are used as outliers. Branches with bootstrap values (based on 500 replicates) of at least 0.95 are shown with asterisks.

Next, genetic breakpoints from the representative consensus sequence were analyzed for each sample using jpHMM-HIV (31) and were highlighted in the HIV-1 genome map. As shown in Fig. 2, subtypes of the *gag p17*, *pol PRRT*, *pol IN*, and *env V3C4* fragments that were determined by jpHMM-HIV were identical to those determined by phylogenetic tree analyses (Fig. 1). The expected breakpoints were located at approximately nt 2263 and nt 3358 (TRN1), nt 1360 (TRN2), nt 6340 and nt 8241 (TRN3), nt 6381 and nt 8296 (TRN4), nt 4230 and nt 5613 (TRN5), nt 4230 and nt 5614 (TRN6), and nt 4282 nt 5064, and nt 9030 (TRN7). As expected, the heterosexual transmission pair (TRN5 and TRN6) had two identical breakpoints. Comparative analysis of genome structures showed that the HIV-1 RFs in TRN3 and TRN4 are new different URFs of subtype B and CRF01_AE. These cases are most likely derived from two unrelated patients, presumably infected in Japan or Thailand according to their clinical records. The genome structures are mosaic and similar to that of CRF15_01B, initially identified in Thailand, or CRF59_01B, identified in northeastern China. Phylogenetic tree analyses indicate that the RFs in TRN3 and TRN4 were not classified in these CRFs (Fig. S4). This classification was also supported by subtyping analyses with the COMET HIV-1 tool (32). The RFs in TRN5 and TRN6, derived from heterosexual transmission partners, showed no identical CRFs in the database. Of note, because all the breakpoints of TRN2 RF sequences obtained from three independent experiments were identical, the RFs were considered not to be artifacts of potential *in vitro* recombination errors during the reverse transcription PCRs (RT-PCRs).

**FIG 3:**
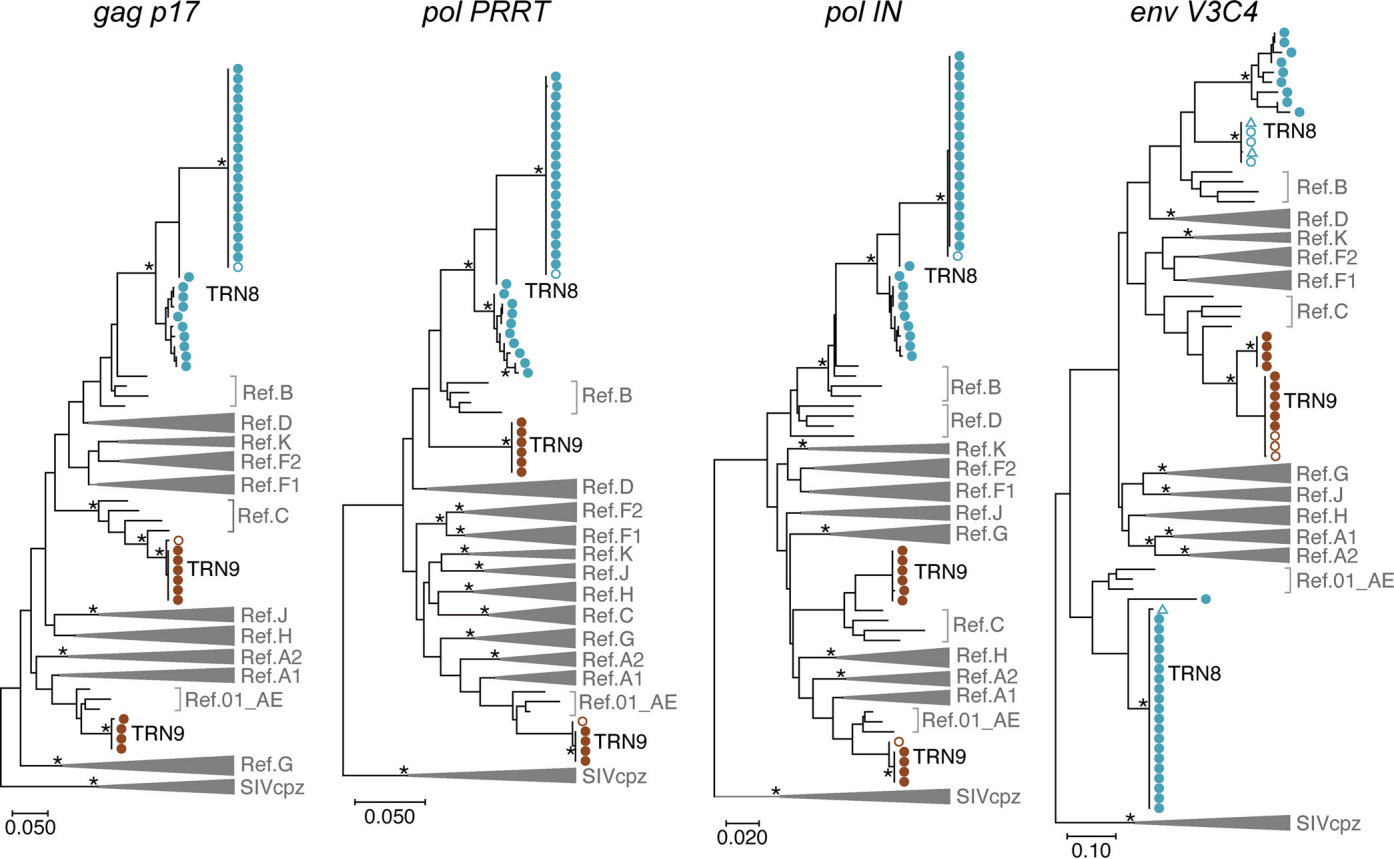
Phylogenies of consensus sequences determined by nanopore sequencing and Sanger methods. Maximum likelihood trees generated with the GTR model are shown for four regions, *gag p17*, *pol PRRT*, *pol IN*, and *env V3C4*, with the nucleotide numbers corresponding to those indicated in Fig. 1. The reference sequences of major HIV-1 subtypes (A1, A2, B, C, D, F1, F2, G, H, J, K, and CRF01_AE) and three SIVcpz strains (LB7, MB66, and MB897) are shown in the trees. Branches with bootstrap values (based on 500 replicates) of at least 0.95 are indicated with asterisks. The viral RNA sequences obtained by Sanger sequencing and nanopore sequencing are indicated with open and closed circles, respectively. The proviral DNA sequences generated by Sanger sequencing are shown with triangles.

**FIG 4:**
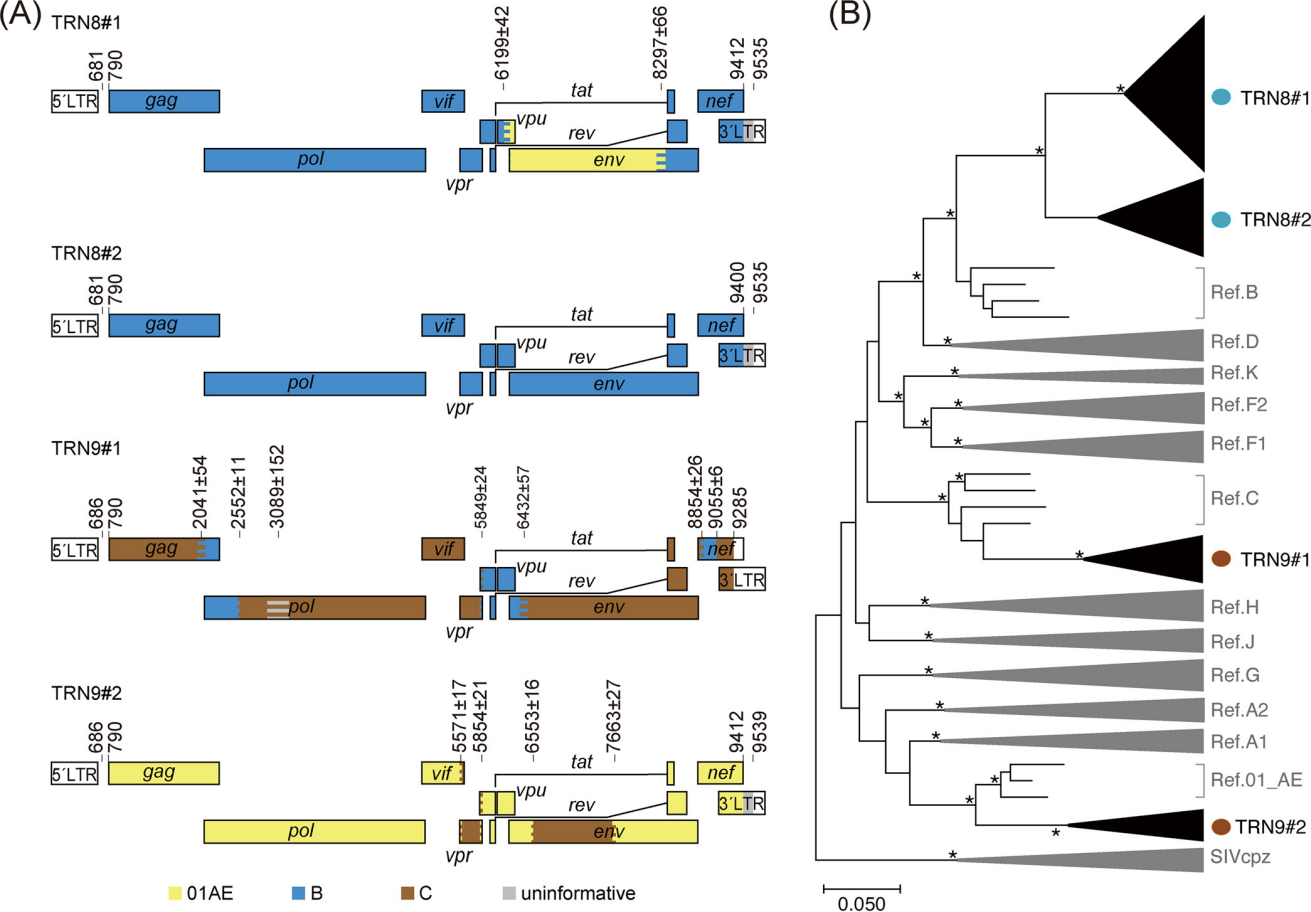
Identification of dual-HIV-1 infection cases. (A) Genome maps of representative sequences obtained by nanopore sequencing. Two different patterns of the genome sequences were identified as dual-infection cases in TRN8 and TRN9. The numbers on each map represent the breakpoints according to the HXB2 reference numbering system. The colored stripes in the maps indicate the intervals where the recombination breakpoints are estimated to be located. (B) Maximum likelihood tree for pangenomic consensus sequences determined by nanopore sequencing and complete genomes of major HIV-1 references. Three sequences of SIVcpz (LB7, MB66, and MB897) are used as outliers. Branches with bootstrap values (based on 500 replicates) of at least 0.95 are indicated with asterisks.

Finally, we examined whether nanopore sequencing is applicable to distinguish different HIV-1 subtypes in dual-infection cases. For this purpose, we performed nanopore sequencing of clinical samples derived from two cases of suspected dual infection. One case (TRN8) had HIV-1 carrying two distinct subtypes in the *env V3C4* region, whereas the other (TRN9) had viruses with two different subtype sequences (AE and C) in the *gag p17*, *pol PRRT*, and *pol IN* regions (Fig. 3 and 4). In TRN8, a maximum of 4 consensus sequences were obtained from every 10 sets of 250 reads using our platform, and thereby a total of 30 consensus sequences were estimated. In TRN9, two haplotypes of the *pol* region fragments that differed in subtypes were identified by nanopore sequencing (Fig. S5). In TRN8, multiple branched variants in the *env V3C4* regions were found in the consensus sequences obtained by nanopore sequencing, although they differed from the sequence variant obtained by Sanger sequencing (Fig. 3, top branches in *env V3C4*). In contrast, two haplotypes of the *pol* region fragments that differed in subtypes were identified by nanopore sequencing (Fig. S5). The results showed that the concordance rates of the mixed bases between Sanger and nanopore sequencing methods were 92% in the *pol PRRT* region and 99% in the *pol IN* region. These data indicate that nanopore sequencing is applicable for distinguishing mixed variants in HIV-1 dual-infection cases.

We counted the number of raw reads corresponding to one phylogenetic cluster in TRN8 and TRN9. In 2,500 raw reads of TRN8, 2,138 (85.5%) and 341 (13.6%) were assigned to the clusters TRN8 no. 1 and no. 2, respectively, whereas the rest of the reads (21 reads, 0.8%) were not mapped to either of the two clusters. In 2,500 reads of TRN9, 1,568 (62.7%) and 932 (37.3%) were assigned to the TRN9 no. 1 and no. 2, respectively. These ratios of nanopore raw reads may reflect to the variants’ ratios from obtained consensus sequences in dual-infection samples.

## DISCUSSION

In this study, we developed a new analytical platform for HIV-1 sequence data obtained by nanopore sequencing. This system includes a selection procedure involving the extraction of end-to-end sequences for target regions, which reduces coverage bias and misalignment risk in the process of consensus sequence inference. In addition, estimation performed 10 times using a different set of 250 sequence reads allowed us to increase the reproducibility of the consensus sequence results as well as the sensitivity of minority variant detection. We found that the error rate of this method was reduced to 0.011 to 0.056%. In addition, we applied this protocol to clinical samples and successfully determined the genotypes of HIV-1 RFs as well as mixed variants in dual-infection cases with sufficient accuracy.

To date, the genetic diversification of HIV-1, including the emergence of new RFs, has gradually increased. Therefore, HIV-1 pangenome sequencing will be recommended to track epidemiological transmission and to identify new RFs (33). In addition, several studies have shown that certain drug resistance-associated mutations are located outside target sequence regions for the HIV-1 drug resistance genotyping test, suggesting that long-read sequencing is required for HIV-1 genotyping analysis. For example, there are reports on certain cleavage-site mutations within Gag and Gag-Pol recognized by viral protease (34, 35), integrase strand transfer inhibitor resistance-associated mutations in the *env* region (36, 37), and the polypurine tract (38, 39). Moreover, a new class of HIV-1 capsid inhibitors, lenacapavir, under clinical development, targets the *gag p24* region (40), which is not currently included in the analysis of viral genome sequences for the HIV-1 drug resistance test. In these circumstances, our new analytical platform for nanopore sequencing will have advantages in determining sequences of the full-length HIV-1 genome efficiently and accurately.

Despite the sufficient accuracy of our nanopore sequencing protocol to assign subtypes, there are two major limitations to HIV-1 genome determination by this nanopore sequencing platform. First, as a common weakness of current nanopore sequencing technology, sequencing errors infrequently occur in homopolymer or tandem repeat regions (30). Because the HIV-1 genome often has such homopolymeric sequences in the *pol* and poly-purine tract regions, genetic analyses at a single-base resolution require extra validations to ensure that there are no unintended insertions and deletions. Second, because minority reads obtained by nanopore sequencing are masked by a major sequence during consensus sequence construction in our protocol, sequence diversities obtained by our protocol might not reflect the original ones, especially for minority HIV-1 variants.

Recently, Wright et al. reported a canonical nanopore sequencing approach after limiting dilution (26), which may be applicable for sequence determination of heterogeneous viral variants. However, the method contains a time-consuming and labor-intensive process of limiting dilution. Therefore, we adopted an alternative approach without such dilution processes, although our approach is designed for an intact HIV-1 genome but not for defective genomes containing large insertions and/or deletions. We performed nanopore sequencing of amplicons that were simply prepared from bulk viral RNA of clinical samples and demonstrated sequences of different subtypes *in silico*. It is intriguing that the two approaches display similar accuracy to estimate consensus sequences of HIV-1 genomes, despite distinct bioinformatics workflows.

In summary, our nanopore sequencing-based platform for HIV-1 genome analysis enables us to determine viral RNA genome sequences in patient plasma and to efficiently distinguish RFs from mixed genotypes in dual-infection samples. Unlike conventional sequence determination by Sanger sequencing, which is a prerequisite for gene fragment cloning or limited dilution, this nanopore sequencing platform is simple and efficient for determining the near-full-length HIV-1 genome. Our developed protocol will be useful for molecular epidemiological surveillance to examine HIV-1 transmission as well as for genotypic tests of HIV-1 drug resistance.

## MATERIALS AND METHODS

### Study samples.

A retrospective study was conducted on HIV-1-infected patients who underwent HIV-1 genotyping for drug resistance testing between October 2016 and July 2020 at the National Hospital Organization Nagoya Medical Center. The study was approved by the ethics committee of the Nagoya Medical Center (approval no. 2010-310) and conducted according to the principles expressed in the Declaration of Helsinki. Genotyping for the HIV-1 drug resistance test was carried out by amplifying cDNA from viral RNA in serum and by Sanger sequencing of four viral gene regions—*gag p17* (corresponding to nt positions 790 to 1185 in the reference HXB2 sequence [GenBank accession no. K03455]), *pol PRRT* (nt positions 2253 to 3269), *pol IN* (nt positions 4230 to 5093), and *env V3C4* (nt positions 7114 to 7589)—as reported previously (17, 18). For the genotyping test of HIV-1 coreceptor tropism, the *env V3C4* sequences in viral RNA as well as proviral DNA were analyzed. Therefore, we also included the *env V3C4* sequences in proviral DNA determined by Sanger sequencing methods in our analysis (quadruplicate per sample). From the genotyping test results (373 cases), we extracted 9 cases with a high viral load in plasma (>1,000 copies/mL) and discordant subtypes between the 4 regions. Their residual blood samples were used for further analysis in this study.

### Viral RNA extraction and DNA preparation.

Viral RNA was extracted from 140 μL of patient plasma using a QIAamp viral RNA minikit (Qiagen) according to the manufacturer’s instructions. Near-full-length cDNA (*gag-nef*; nt positions 681 to 9556, according to the position numbering of the reference HXB2 strain) or two overlapping half-length cDNAs (*gag-in*, nt 681 to 5219, and *in-nef*, nt 4146 to 9556) of HIV-1 were prepared from the RNA by one-step RT-PCR followed by nested PCR using a PrimeScript II high fidelity one-step RT-PCR kit (TaKaRa Bio) and PrimeSTAR GXL DNA polymerase (TaKaRa Bio). The primer sets (Table S1) used in this study were optimized for HIV-1 full-genome amplification as previously reported (41) and might be key to obtaining full-genome and/or half-genome DNA fragments of HIV-1 by RT-nested PCR. The nested PCR was performed in a total reaction volume of 50 μL per sample, containing distilled water, 5× PrimeSTAR GXL buffer, forward and reverse primers (0.25 μM each), a deoxynucleoside triphosphate (dNTP) mixture (2.5 mM each), high-fidelity PrimeSTAR GXL DNA polymerase (1.25 U/reaction), and cDNA. The PCR cycling conditions were as follows: (i) 1 min at 98°C for denaturation and DNA polymerase activation, (ii) 30 cycles of denaturation (10 sec at 98°C), annealing (15 sec at 55–60°C), and elongation (1.5 to 5 min at 68°C), and (iii) a final elongation step for 5 min at 68°C. The amplified DNA fragments were analyzed through 0.7% agarose gel electrophoresis with ethidium bromide staining.

### Nanopore sequencing.

DNA libraries for nanopore sequencing were prepared according to the protocol outlined by Oxford Nanopore Technology (ONT) (ACDE_9064_v109_revP_14Aug2019) and quantitated using the Qubit double-stranded DNA (dsDNA) highly selective assay kit (Thermo Fisher Scientific). For all samples, 100 to 200 fmol of amplicon DNA was mixed with NEBNext FFPE DNA repair mix and NEBNext Ultra II end repair/deoxyribosyladenine (dA)-tailing module reagents (New England Biolabs) and incubated at 20°C for 5 min, followed by inactivation at 65°C for 5 min. The end-repaired DNA amplicons were purified with AMPure XP beads (Beckman Coulter) and were barcoded with unique adapter indexes of the Nanopore native barcoding expansion kit (EXP-NBD104) (ONT). The resultant barcoded DNA amplicons were pooled and then loaded into a port on the R9.4.1 flow cell (ONT). Nanopore sequencing data were obtained through an Oxford Nanopore MinION Mk1B device and MinKNOW software (ONT). A brief flow chart of the nanopore sequencing protocol used to obtain HIV-1 genome sequences is illustrated in Fig. S1.

### Consensus sequence determination.

Consensus sequences for each HIV-1 sample were individually determined. Briefly, DNA bases were called from raw signal data in FAST5 format files, and the barcoded portions were trimmed using Guppy v3.6 (ONT). The sequenced raw reads were output in FASTQ format. Next, the sequences that were the −500 to +1,000 nt of their expected length were extracted using NanoFilt v2.6.0 (42) and then used to determine draft consensus sequences with Canu v2.0 (43, 44). Finally, to construct polished consensus sequences for each sample, the draft sequences were error-corrected with racon v1.4.17 (45) and medaka v1.0.1 (https://github.com/nanoporetech/medaka). In this study, consensus sequences were repeatedly created from 10 different sets of 250 raw reads. In the event that different consensus sequences were obtained from each set of reads, all the consensus sequences determined by these methods were used for subsequent analyses. Additionally, in an event when no draft sequences for one set of the 250 reads were obtained even through a maximum of 10 distinct tests using Canu, we omitted the analysis data of the particular set. For construction of consensus sequences from two overlapping fragments of the HIV-1 genome, consensus sequences of the two fragments were aligned and manually connected to form one near-full-length sequence using MEGA X (46).

### Phylogenetic analysis and RF characterization.

Phylogenetic relationships among the virus samples were examined using the determined consensus sequences. A multiple-sequence alignment was performed with MAFFT v7.372 (47) with HIV-1 reference sequences of each subtype (A1, A2, B, C, D, F1, F2, G, H, J, K, and CRF01_AE) and three simian immunodeficiency syndrome from chimpanzees (SIVcpz) sequences (GenBank no. DQ373064, DQ373063, and EF535994 for SIVcpzLB7, SIVcpzMB66, and SIVcpzMB897, respectively), which were obtained from the HIV sequence database at the Los Alamos National Laboratory (4). Maximum likelihood phylogenetic trees were constructed from alignments of the sequences using MEGA X software (46) with 500 bootstrap replicates and the generalized time-reversible (GTR) model. Genetic distances were calculated based on all of the alignment positions. Genetic organization and recombination breakpoints in HIV-1 sequences were analyzed by using the jpHMM-HIV program (31) with its default settings. Subtypes identified by phylogenetic analysis were complementarily confirmed using the HIV-1 subtype classification tool COMET HIV-1 (32). For the TRN2, the sequencing analyses were performed independently three times to verify the breakpoints.

### Data availability.

The nanopore sequencing data obtained in this study are available in the DNA Data Bank of Japan (DDBJ) Sequenced Read Archive under BioProject accession ID no. PRJDB13369.
