# Supplementary material for: Nanopore Sequencing for Characterization of HIV-1 Recombinant Forms
Source: Microbiol Spectr. 2022 Jul 27;10(4):e01507-22. doi: 10.1128/spectrum.01507-22 (PMC9431566; doi:10.1128/spectrum.01507-22)
Supplement: Supplemental file 1 — Supplemental material. Download spectrum.01507-22-s0001.pdf, PDF file, 1.8 MB [file spectrum.01507-22-s0001.pdf]

Supplemental Material

Nanopore sequencing for characterization of HIV-1 recombinant forms

Mikiko Mori[a,b], Hirotaka Ode[a], Mai Kubota[a], Yoshihiro Nakata[a,b], Takaaki Kasahara[a,b], Urara Shigemi[a], Reiko Okazaki[a], Masakazu Matsuda[a], Kazuhiro Matsuoka[a], Atsuko Sugimoto[a], Atsuko Hachiya[a], Mayumi Imahashi[a], Yoshiyuki Yokomaku[a], and Yasumasa Iwatani[a,b]#

[a]Clinical Research Center, National Hospital Organization Nagoya Medical Center, Nagoya, Japan

[b]Division of Basic Medicine, Nagoya University Graduate School of Medicine, Nagoya, Japan

TABLE S1. List of primer sets for genome amplification by RT-PCR (1st) and nested PCR (2nd).

			sequences (5′ to 3′)	length (base)	Position number of nucleotide (5′ to 3′)
gag-nef	1st	F *	ATCTCTAGCAGTGGCGCCCGAACAG	25	625-649
	2nd	F	CTCTCTCGACGCAGGACTCGGCTTG	25	681-705
	1st	R	CACTCAAGGCAAGCTTTATTGAGGC	25	9,630-9,606
	2nd	R	GGTCTAACCAGAGAGACCCAGTACAG	26	9,556-9,531
gag-in	1st	F	ATCTCTAGCAGTGGCGCCCGAACAG	25	625-649
	2nd	F	CTCTCTCGACGCAGGACTCGGCTTG	25	681-705
	1st	R	CCTGTATGCAGACCCCAATATG	22	5,264-5,243
	2nd	R	CCTAGTGGGATGTGTACTTCTGAACTTA	28	5,219-5,192
in-nef	1st	F	CAGACTCACAATATGCATTAGG	22	4,039-4,060
		F †	CAGACTCACAGTATGCATTAGG	22	4,039-4,060
	2nd	F	CTGGCATGGGTACCAGCACACAA	23	4,146-4,168
	1st	R	CACTCAAGGCAAGCTTTATTGAGGC	25	9,630-9,606
	2nd	R	GGTCTAACCAGAGAGACCCAGTACAG	26	9,556-9,531

* F and R denote forward and reverse primers, respectively.

†The primer was newly designed in this study.

The numbers represent the nucleotide positions in the HXB2 reference sequence.

Supplementary Figures

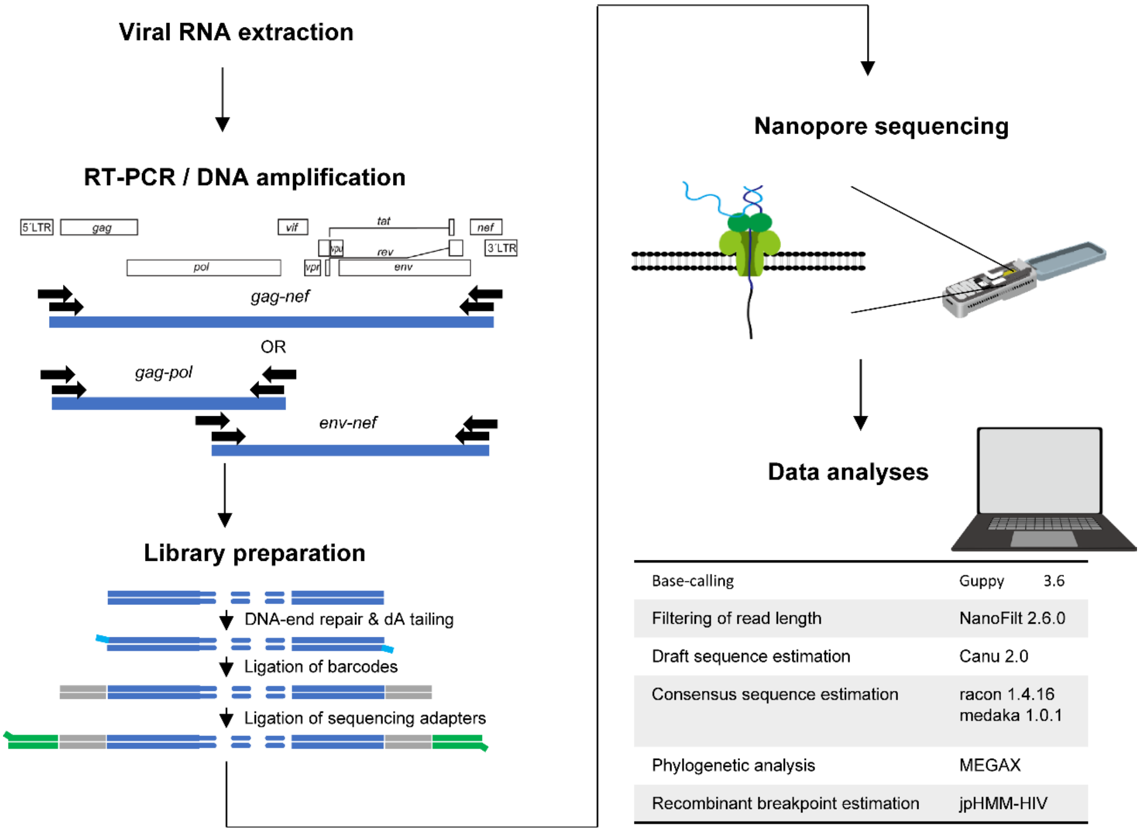

FIG S1 A flowchart of the nanopore sequencing protocol applied to the near-full-length HIV-1 genome in this study.

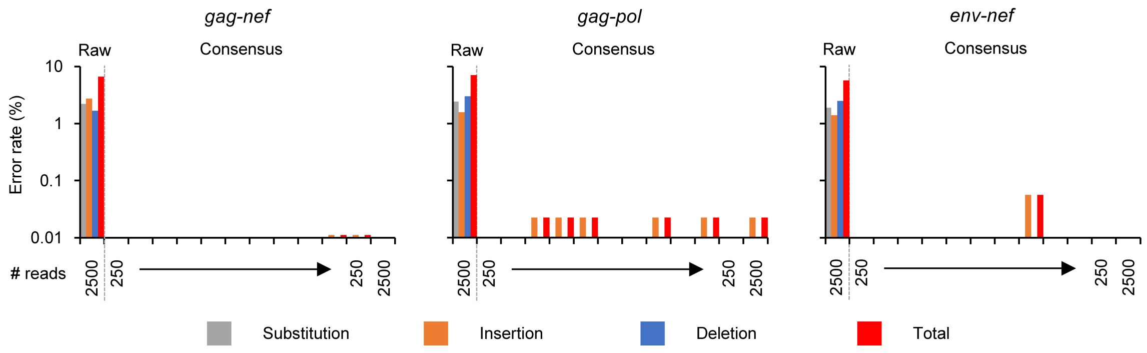

FIG S2 Error rate (%) among raw reads and consensus sequences generated by nanopore sequencing. The proviral DNA, pNL4-3, was used for analysis. Individual error patterns (substitutions, insertions, deletions, and their total) are colored. Consensus sequences were estimated repeatedly from 10 different sets of 250 reads or from all reads (2,500 reads). No bar represents no error.

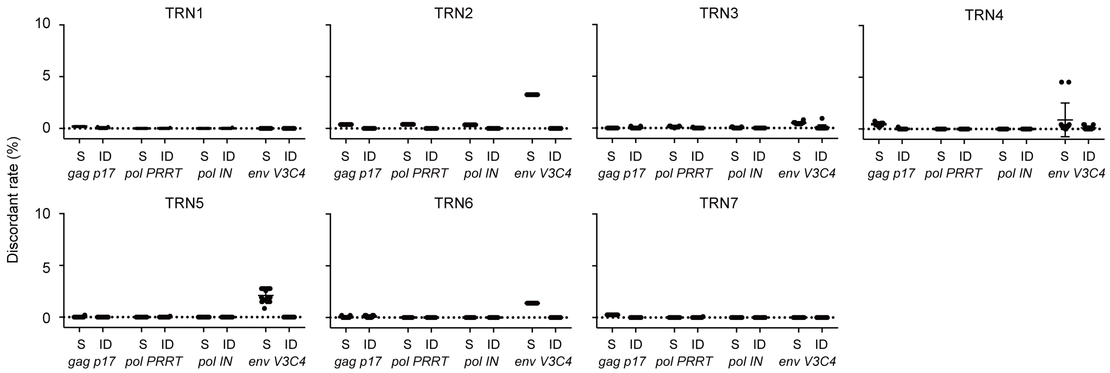

FIG S3 Ratio of mismatched bases between Sanger and nanopore sequencing. The viral genome sequences in the *gag p17* (positions 790 to 1,185 relative to the reference HXB2), *pol PRRT* (2,253 to 3,269), *pol IN* (4,230 to 5,093), and *env V3C4* (7,114 to 7,589) regions were compared. The ratios at the nucleotide positions are plotted with dots. Horizontal lines indicate the means with SDs shown as error bars. S, substitution. ID, insertion or deletion.

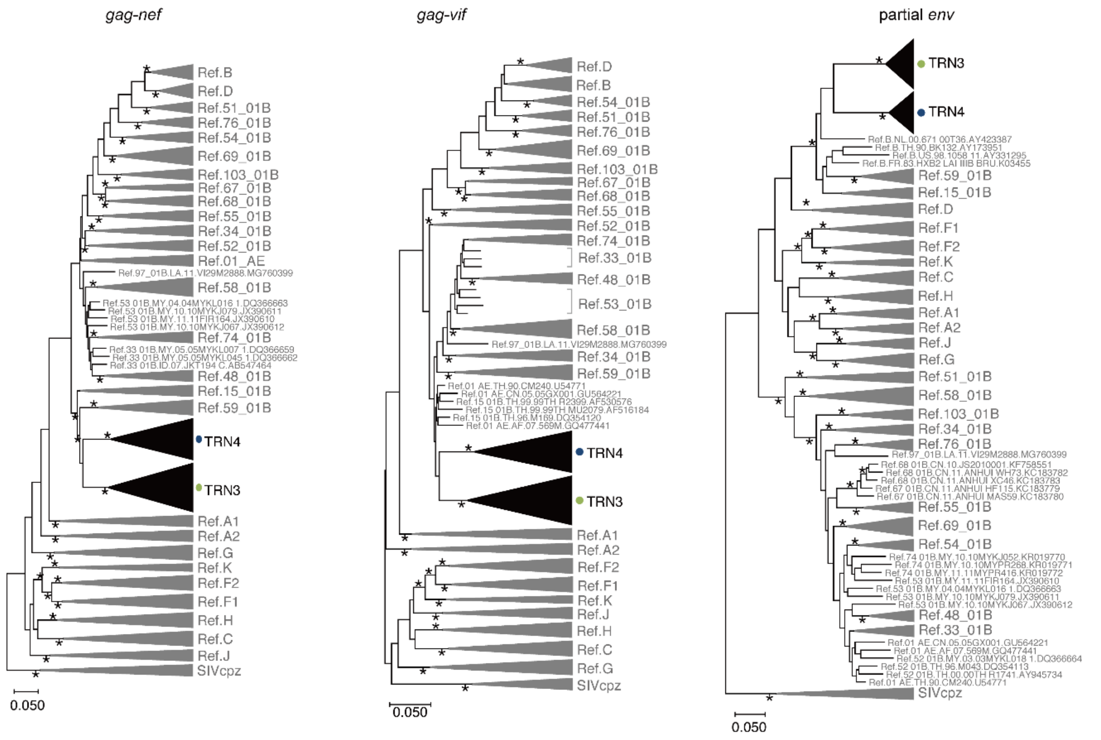

FIG S4 Phylogenetic relationships of the recombinant forms between subtype B and CRF01_AE. Pangenomic sequences and coding sequences of the *gag-vif* (positions 790 to 5,619 nt) and *env* (positions 6,300 to 8,200 nt) regions were analyzed. A maximum likelihood tree was constructed. Three SIVcpz sequences (GenBank IDs: DQ373064, DQ373063, and EF535994) are used as outliers. Branches with bootstrap values (based on 500 replicates) of at least 0.95 are highlighted with asterisks.

pol PRRT

HXB2	2285	2288	2294	2295	2300	2303	2306	2307	2312	2313	2323	2358	2361	2368	2373	2388	2424	2427	2433	2436	2437	2442	2443	2455	2459
Sanger	Y	M	R	R	R	R	R	Y	R	R	Y	Y	R	M	R	R	R	R	D	R	R	Y	Y	K	M
TRN9#1	T	C	G	A	G	G	A	T	A	G	T	C	G	A	G	A	A	G	A	A	G	C	C	T	C
TRN9#2	C	A	A	G	A	A	G	C	G	A	C	T	A	C	A	G	G	A	T	G	A	T	T	T	A
	✓	✓	✓	✓	✓	✓	✓	✓	✓	✓	✓	✓	✓	✓	✓	✓	✓	✓		✓	✓	✓	✓		✓

HXB2	2461	2464	2467	2478	2479	2482	2483	2512	2513	2519	2536	2551	2557	2569	2583	2584	2587	2605	2620	2633	2639	2646	2656	2657	2659
Sanger	W	R	Y	Y	R	R	R	R	M	M	Y	Y	Y	M	M	R	M	M	R	R	R	R	Y	R	M
TRN9#1	T	A	C	T	A	A	A	A	A	C	T	T	C	A	A	G	C	C	A	A	G	G	C	A	C
TRN9#2	A	G	T	T	G	G	G	A	C	A	C	C	T	C	C	A	A	A	G	G	A	A	T	G	A
	✓	✓	✓		✓	✓	✓		✓	✓	✓	✓	✓	✓	✓	✓	✓	✓	✓	✓	✓	✓	✓	✓	✓

HXB2	2680	2693	2694	2723	2730	2738	2744	2747	2750	2754	2756	2759	2762	2768	2786	2789	2803	2807	2810	2840	2849	2861	2877	2880	2889
Sanger	R	Y	W	Y	R	Y	R	R	R	R	Y	W	R	R	R	R	Y	R	Y	Y	W	R	R	R	R
TRN9#1	A	T	A	C	A	C	A	G	A	A	T	T	G	A	G	A	C	A	T	C	T	G	A	G	G
TRN9#2	A	C	T	T	G	T	G	A	G	G	C	A	A	G	A	G	C	G	C	T	A	A	G	A	A
		✓	✓	✓	✓	✓	✓	✓	✓	✓	✓	✓	✓	✓	✓	✓		✓	✓	✓	✓	✓	✓	✓	✓

HXB2	2907	2914	2920	2925	2928	2931	2961	2967	2976	2979	2982	2997	3000	3021	3024	3033	3036	3037	3050	3060	3063	3071	3073	3074	3082
Sanger	Y	K	R	Y	R	R	Y	R	R	Y	R	R	W	W	R	Y	R	W	W	R	Y	R	R	M	R
TRN9#1	T	T	G	C	G	A	T	A	G	T	G	A	T	A	A	T	A	A	T	G	T	A	A	C	A
TRN9#2	C	G	A	T	A	G	C	G	A	C	A	G	A	T	G	C	G	T	A	A	C	G	A	A	G
	✓	✓	✓	✓	✓	✓	✓	✓	✓	✓	✓	✓	✓	✓	✓	✓	✓	✓	✓	✓	✓	✓		✓	✓

HXB2	3085	3091	3094	3103	3115	3136	3145	3157	3169	3173	3187	3199	3211	3214	3223	3229	3235	3238	3247
Sanger	S	Y	Y	Y	R	R	K	R	R	S	S	Y	R	R	R	K	Y	K	R
TRN9#1	C	C	C	C	G	G	G	A	G	C	G	C	G	A	A	T	T	T	G
TRN9#2	G	T	T	T	A	A	T	A	A	G	C	T	A	G	G	G	C	T	A
	✓	✓	✓	✓	✓	✓	✓		✓	✓	✓	✓	✓	✓	✓	✓	✓		✓

pol IN

HXB2	4233	4241	4250	4263	4271	4281	4312	4320	4321	4324	4341	4344	4354	4356	4379	4400	4419	4427	4430	4433	4434	4436	4447	4448	4456
Sanger	Y	R	R	R	R	Y	Y	Y	R	R	R	Y	M	R	Y	Y	Y	Y	M	Y	Y	R	R	R	R
TRN9#1	C	A	A	G	A	C	C	C	A	G	G	T	C	G	C	C	T	T	C	T	T	G	G	A	A
TRN9#2	T	G	G	A	G	T	T	T	G	A	A	C	A	A	T	T	C	C	A	C	C	A	A	G	G
	✓	✓	✓	✓	✓	✓	✓	✓	✓	✓	✓	✓	✓	✓	✓	✓	✓	✓	✓	✓	✓	✓	✓	✓	✓

HXB2	4468	4471	4483	4486	4495	4499	4502	4517	4520	4565	4572	4577	4586	4590	4591	4592	4604	4634	4635	4636	4637	4638	4642	4649	4655
Sanger	Y	R	Y	R	R	Y	Y	R	R	M	R	Y	Y	M	S	Y	Y	M	R	R	K	R	R	M	R
TRN9#1	T	A	C	G	G	C	T	A	A	C	G	T	T	A	G	T	T	A	G	G	G	A	A	C	A
TRN9#2	C	G	T	A	A	T	C	G	G	A	A	C	C	C	C	C	C	C	A	A	T	G	G	A	G
	✓	✓	✓	✓	✓	✓	✓	✓	✓	✓	✓	✓	✓	✓	✓	✓	✓	✓	✓	✓	✓	✓	✓	✓	✓

HXB2	4658	4679	4695	4704	4716	4719	4737	4746	4776	4815	4834	4874	4890	4921	4936	4938	4954	4987	4999	5005	5011	5023	5035	5041	5043
Sanger	Y	R	Y	R	R	Y	W	R	R	R	R	Y	Y	R	M	K	M	W	K	Y	R	R	R	Y	R
TRN9#1	T	G	C	A	A	T	T	G	A	G	G	C	C	A	C	G	C	A	G	C	G	G	A	T	A
TRN9#2	C	A	T	G	G	C	A	A	G	A	A	T	T	G	A	T	A	A	T	T	A	A	G	C	G
	✓	✓	✓	✓	✓	✓	✓	✓	✓	✓	✓	✓	✓	✓	✓	✓	✓		✓	✓	✓	✓	✓	✓	✓

HXB2	5068	5070	5099
Sanger	Y	M	R
TRN9#1	C	C	G
TRN9#2	T	A	A
	✓	✓	✓

FIG S5 Detection of haplotype sequences in the *pol* region in TRN9 by nanopore sequencing. Two or more mixed bases at the 119th position in the *pol PRRT* gene (1,017 nt) and at the 78th position in the *pol IN* gene (864 nt) were detected. Subsequent analysis by nanopore sequencing revealed two haplotype sequences in the regions. Major base contents of the positions (92% and 99% at positions 110 nt and 77 nt, respectively) were separately assigned to the two haplotype sequences (marked by "✓"). Minor base contents (gray background) were situated where the bases were consistently assigned between the two haplotype sequences through both nanopore sequencing and Sanger sequencing. D = A, G, or T.
